# Investigating the mechanism of rough phenotype in a naturally attenuated *Brucella* strain: insights from whole genome sequencing

**DOI:** 10.3389/fmed.2024.1363785

**Published:** 2024-04-22

**Authors:** Wendong Han, Dong Wei, Zhiping Sun, Di Qu

**Affiliations:** ^1^BSL-3 Laboratory of Fudan University, Key Laboratory of Medical Molecular Virology (MOE/NHC/CAMS), Department of Medical Microbiology and Parasitology, School of Basic Medical Sciences, Shanghai Medical College, Fudan University, Shanghai, China; ^2^Division of Tuberculosis Vaccines and Allergen, National Institute for Food and Drug Control, Beijing, China; ^3^Shanghai Institute of Infectious Diseases and Biosecurity, Shanghai Medical College, Fudan University, Shanghai, China

**Keywords:** *Brucella melitensis*, *de novo* sequencing, comparative genomic analysis, attenuated strain, LPS

## Abstract

**Objective:**

Brucellosis, a significant zoonotic disease, not only impacts animal health but also profoundly influences the host immune responses through gut microbiome. Our research focuses on whole genome sequencing and comparative genomic analysis of these *Brucella* strains to understand the mechanisms of their virulence changes that may deepen our comprehension of the host immune dysregulation.

**Methods:**

The *Brucella melitensis* strain CMCC55210 and its naturally attenuated variant CMCC55210a were used as models. Biochemical identification tests and *in vivo* experiments in mice verified the characteristics of the strain. To understand the mechanism of attenuation, we then performed *de novo* sequencing of these two strains.

**Results:**

We discovered notable genomic differences between the two strains, with a key single nucleotide polymorphism (SNP) mutation in the *manB* gene potentially altering lipopolysaccharide (LPS) structure and influencing host immunity to the pathogen. This mutation might contribute to the attenuated strain's altered impact on the host's macrophage immune response, overing insights into the mechanisms of immune dysregulation linked to intracellular survival. Furthermore, we explore that manipulating the Type I restriction-modification system in *Brucella* can significantly impact its genome stability with the DNA damage response, consequently affecting the host's immune system.

**Conclusion:**

This study not only contributes to understanding the complex relationship between pathogens, and the immune system but also opens avenues for innovative therapeutic interventions in inflammatory diseases driven by microbial and immune dysregulation.

## 1 Introduction

*Brucella*, a genus of Gram-negative facultative intracellular bacteria, can survive and replicate in host macrophages. *Brucella* infection may result in host brucellosis, a disease characterized by abortion and sterility in livestock, as well as undulant fever, arthritis, and neuro-inflammations in humans. With 500,000 human annual cases globally reported (1), the World Health Organization ranks *brucellosis* as “neglected zoonosis” (2). *Brucellosis* can be transmitted to humans through contact with aborted animal fetuses, inhalation of bacterial aerosols, or consumption of contaminated milk and dairy products. There are currently 12 *Brucella* species identified in nature; of them, *Brucella melitensis* (*BM*)*, Brucella abortus* (*BA*), *Brucella suis* (*BS*), and *Brucella canis* (*BC*) are the significant pathogens resulting in public health challenges and economic implications.

*Brucella* spp. are known to enter the body primarily through the gastrointestinal tract, where they encounter various barriers and immune responses. Upon reaching the gastrointestinal tract, *Brucella* spp. face the acidic pH of the stomach and the antimicrobial action of bile salts in the small intestine. Crossing the intestinal epithelial barrier involves interactions with M cells, which are specialized for antigen uptake. The gut-associated lymphoid tissue (GALT) plays a crucial role in the immune response to *Brucella*, with dendritic cells and macrophages in the lamina propria participating in antigen presentation and activation of T and B lymphocytes. Furthermore, the gut microbiome's role in systemic immunity is increasingly recognized, with 70%−80% of immune cells being present in the gut. Nutrition has a significant impact on the composition of the gut microbiota and, subsequently, on the immune system, highlighting the potential of dietary strategies in enhancing immune responses and treatment outcomes for infectious diseases.

Vaccination is a critical, general, and cost-effective approach for *Brucellosis* control and eradication compared with specific anti-*Brucella* drugs. Still, there has been no successful vaccine for human *brucellosis* till now (3). Therefore, human brucellosis can be controlled only by decreasing brucellosis in animals. The currently applied animal vaccines are RB51, S19, Rev.1, S2, and SR82. For example, the strain *BA* RB51, approved in 1996 for cattle, is a rough, genetically stable, attenuated, spontaneous mutant of virulent *BA* 2308. The rough phenotype of RB51 is due to the mutation of *wboA* that encodes a glycosyltransferase, a key protein of O antigen synthesis, disrupted by an IS711 element (4). Widespread use of commercial vaccines has dramatically reduced the incidence of *brucellosis*, but these vaccines still have disadvantages such as residual virulence, longer survival periods in natural hosts, antibiotic resistance, and interference with conventional serological tests. Whole genome sequencing and genome comparative analysis of spontaneous attenuated strains offer a pathway for developing ideal vaccines against *brucellosis* and understanding the mechanisms of the mutant function.

In this study, we obtained a spontaneously rough strain named CMCC55210a, which evolved from the smooth strain *BM* CMCC55210. Subsequently, biochemical identification tests and *in vivo* experiments in mice verified the strain to be stable and attenuated. To understand the mechanism of attenuation, we then performed *de novo* sequencing of these two strains. Through comparative genomic analysis, a possible mechanism of virulence reduction from single nucleotide mutation in phosphomannomutase encoding gene *manB* was shown at the gene level. Further studies should be performed to evaluate the immunogenicity, safety, and protection of strain CMCC55210a as a vaccine candidate.

## 2 Materials and methods

### 2.1 *Brucella* strains and culture

*Brucella melitensis* is a Gram-negative coccobacillus in the family *Brucellaceae* (class Alphaproteobacteria). There are three biovars, 1–3. *Brucella abortus* and *B. suis* have been found occasionally in small ruminants, but clinical cases caused by these organisms seem to be rare. *Brucella melitensis* is zoonotic. Small ruminants often acquire *B. melitensis* by contact with organisms in vaginal discharges and birth products (e.g., placenta, fetus, and fetal fluids). Most animals are thought to become infected by ingestion and through the oronasal and conjunctival mucosa, but this organism can also be transmitted venereally and through broken skin. *Brucella* spp. are readily killed by most commonly available disinfectants, including hypochlorite solutions, sodium hydroxide, quaternary ammonium compounds, 70% ethanol, isopropanol, iodophors, phenolic disinfectants, formaldehyde, glutaraldehyde, and xylene.

A smooth virulence strain of *BM* CMCC55210 was provided by the National Center for Medical Culture Collections (CMCC) of China, originally from the Animal Health and Veterinary Laboratories Agency of the UK. *Brucella* phages Tb, Wb, Fi, and BK2 were from the China National Institutes for Food and Drug Control.

*Brucella* strains were cultivated in tryptic soy broth (TSB; tryptic soy broth, BD Difco, USA) or on tryptic soy agar (TSA) plates (tryptic soy agar, BD Difco, USA) and stored in TSB with 20% (v/v) glycerol (Sigma-Aldrich, USA) at −80°C. The bacteria were cultured in the biosafety level 3 laboratory of Fudan University.

### 2.2 Screen of natural rough mutant

Frozen *Brucella* strains were removed from −80°C and completely thawed at room temperature. Ten microliter of the strain *BM* CMCC55210 stock solution was inoculated by spreading on TSA and incubated at 37°C for 3 days. Bacteria were harvested and resuspended in normal saline (NS, 0.9% NaCl) to obtain a bacterial solution at a final concentration of ~1.0 × 10^9^ colony-forming units (CFU)/ml via turbidimetry (standard turbidimetric tubes manufactured by the China Academy of Food and Drug Administration, No. 230021-201354). Slide agglutination assays were used to distinguish between smooth and rough strains for screening natural rough mutants (5).

#### 2.2.1 Mutation test by slide agglutination assays

Two hundred microliter of 10^3^ CFU/ml *BM* CMCC55210 suspension was inoculated on TSA plates, spread well, and incubated at 37°C for 3 days. Single-colony colonies were picked and inoculated onto a coverslip, 50 μl of acridine yellow hydrochloride solution (1:500, Sigma-Aldrich, USA) was added, the coverslip was covered and then gently ground. The colonies observed visually with noticeable agglomerated particles or flocculent particles were judged as rough mutants. The remaining colonies of the mutant were picked and inoculated on TSA plates and incubated at 37°C for 2 days. Then, the bacteria were harvested by scraping the moss, added to 1 ml of preservation solution (TSB solution containing 20% glycerol), and then frozen at −80°C in a refrigerator. Crystal violet staining was performed to further verify the rough phenotype of the selected mutant strains (6).

#### 2.2.2 Validation by crystal violet staining

One hundred microliter of 10^3^ CFU/ml *BM* CMCC55210a rough bacterial suspension and *BM* CMCC55210 smooth bacterial suspension were inoculated on TSA plates separately, spread well, and incubated for 3 days at 37°C. Subsequently, 2 ml of staining solution [0.05% crystal violet (Sigma-Aldrich, USA), 25% methanol] was poured over the plates. After 15 s, the staining solution was discarded and poured into a disinfectant (75% ethanol). In contrast to the light purple background, smooth colonies were stained a translucent light blue-green, whereas rough colonies showed red to blue-red. Following the staining process, the peripheral colors of smooth colonies progressively became deeper blue-violet, but the uniform coloration of rough colonies was maintained for 4 days.

### 2.3 The selected rough mutant confirmed as *Brucella melitensis*

To confirm whether the rough mutant originated from the smooth *Brucella melitensis* CMCC55210, the dye sensitivity, H_2_S production, and phage lysis tests were carried out.

#### 2.3.1 Dye sensitivity (basic fuchsin and thionin)

The bacterial suspension of smooth strain and selected natural mutant strain was inoculated on TSA medium containing basic fuchsin and thionin (1:50,000 w/v, Sigma-Aldrich, USA), respectively, and then incubated at 37°C for 3 days. Bacteria growth was determined by colony counting. Both *BM* strains should grow well on the TSA plates, adding basic fuchsin and thionin as normal TSAs.

#### 2.3.2 H_2_S production

The strains *BM* CMCC55210 and CMCC55210a were inoculated on TSA plates. Lead acetate test papers (Sigma-Aldrich, USA) were placed on the surface of the agar and co-incubated at 37°C. The color of the test papers was checked after 2 days. The paper would turn black when hydrogen sulfide is produced. The cultivation of *Brucella melitensis* should be free of hydrogen sulfide production.

#### 2.3.3 Phage lysis of the strains

Phage propagation and titration were those described in previous references (7). One hundred microliters of 10^9^ CFU/ml *BM* CMCC55210 and CMCC55210a were inoculated on the TSA medium and distributed evenly. Each plate was dripped with 5 μl of *Brucella* bacteriophage Tb, Wb, Fi, and BK2 at a routine test dilution (RTD) concentration. After incubating at 37°C for 2 days, the plaques by phage lysis were visually determined. Phages Tb, Wb, and Fi were unable to lyse *Brucella melitensis*, and no phage plaque was present on the plates. Phage BK2 was capable of lysing *Brucella melitensis*, and neatly edged, translucent plaques were presented.

### 2.4 Bacteria virulence test in BALB/c mice

#### 2.4.1 Animals

Female BALB/c mice (~42 days old), purchased from Slack Laboratory Animal Co., Ltd (Shanghai, China), were kept in an individually ventilated cage (IVC) rack system of the ABSL-3 laboratory of Fudan University. Animal experiments were reviewed and approved by the Animal Welfare and Ethics Committee of the School of Basic Medical Science of Fudan University (Approval No. 20201105-001).

#### 2.4.2 Virulence of Brucella mutant strain in mice

The strains *BM* CMCC55210 and CMCC55210a were cultured on TSA for 3 days, and the bacterial suspension was harvested in NS and adjusted to 10^9^ CFU/ml as previously. Mice were challenged by intraperitoneal injection with 100 μl bacterial suspension of CMCC55210 strain and CMCC55210a strain. The number of mice in the negative control group was injected intraperitoneally with 100 μl of NS. Actual infection doses as above were further confirmed by serial dilution, culture, and CFU counting. At 2-, 14-, 28-, 42-, and 56-day post-infection, 14 mice (including six of the CMCC55210 group, six of the CMCC55210a group, and two of the NS group) were weighed and euthanized with an overdose of sodium pentobarbital (200 mg/kg body mass) and then immersed in 75% alcohol for 3 min. Afterward, the spleens were aseptically obtained, weighed, examined for gross pathology, photographed, and then homogenized in 1 ml PBS buffer. The spleen homogenates were diluted 10-fold with 1 × PBS in sequence and plated on TSA to determine the spleen bacteria load by counting CFU. The splenomegaly index was performed as spleen/mouse weight × 100%.

### 2.5 Whole-genome *de-novo* sequencing, assembly

The genomic DNA of *Brucella melitensis* CMCC55210 and CMCC55210a was extracted with QIAamp DNA Mini Kit (QIAgen, Germany) according to the manual in the BSL-3 laboratory. DNA concentration was measured using a Nanodrop2000 (Thermo Scientific, Wilmington, USA), and the quality was evaluated by 1.5% agarose gel electrophoresis.

Genome *de novo* sequencing was performed by the Shanghai Majorbio Bio-pharm Biotechnology Company (China). In brief, paired-end kits and mate-pair kits from Illumina were used to construct PE libraries (average insert sizes of ~170, 250, 500, and 800 bp) and MP libraries (average insert sizes of 2, 5, 10, 20, and 40 kb). DNA sequencing was performed on the HiSeq 2500 platform (Illumina). Ten- and 20- kilobyte libraries were constructed for PacBio sequencing on a PacBio RS II sequencer (Pacific Biosciences). The genome assembly was based on both Illumina sequencing and PacBio SMRT sequencing data. The hierarchical genome assembly process (HGAP4, Pacific Biosciences) was followed for assembling continuous long reads (CLR) from PacBio sequencing. Then, a four-round iterative error correction process was performed with clean data from Illumina sequencing to increase assembly accuracy. Finally, we generated a 2-chromosome circular gapless genome sequence of *BM* CMCC55210 and CMCC55210a. For *BM* CMCC55210, 162,199 clean reads with an average length of 7126 bp and an N50 size of 2,117,097 bp (chr1) and 1,177,809 bp (chr2) were generated. For the *BM* CMCC55210a strain, 113,408 clean reads with an average length of 7,016 bp and an N50 size of 2,117,101 bp (chr1) and 1,177,797 bp (chr2) were generated. The genome sequences of *BM* CMCC55210 (the accession number: CP082109 and CP082110) and CMCC55210a (the accession number: CP082107 and CP082108) were deposited in the NCBI database, respectively.

### 2.6 Bioinformatics analysis of genomes

The coding genes for the genomes of *BM* CMCC55210 and CMCC55210a were predicted by GeneMarkS (8) (ver4.17). The protein sequences of predicted coding genes were aligned using DIAMOND (9) (ver2.0.1), and the selected proteins for annotation were based on gene matching (*e*-values ≤1e-5) and the alignment with the highest score (identity ≥40%, coverage ≥40%). Genomic islands (GIs) were predicted by IslandViewer 4 (10). PhiSpy (11) (ver3.4) was used to predict prophage loci. The general functional pathway analysis was performed with Gene Ontology (GO enrichment analysis). Kyoto encyclopedia of genes and genomes (KEGG) and COG (clusters of orthologous groups) were used for predicting gene functions. The sequencing maps were displayed using Circos software (ver0.64) after combining the prediction results of the encoded genes.

### 2.7 Comparative genome analysis

The average nucleotide identity between *BM* CMCC55210 and CMCC55210a was calculated by JSpeciesWS (12). The progressive Mauve program (ver2.4.0) and MUMmer3 (13) were used to perform whole genome alignments and comparisons. The genomic variations, including insertions (Ins), deletions (Dels), and nucleotide mutations of *BM* CMCC55210a compared with *BM* CMCC55210, were analyzed and rechecked manually using Basic Local Alignment Search Tool (BLAST, ver2.8.1). Nucleotide BLAST and Protein BLAST were used for alignment analysis of DNA sequence and protein sequence separately. Using the AlignX program (Vector NTI, version 11.5.1), each gene's coding sequences (CDSs) and protein sequences in the three samples were compared, analyzed, and visualized. Combined with the protein functional domains predicted by the Simple Modular Architecture Research Tool (SMART) database (14), the impact of the mutation on gene function was determined.

### 2.8 Evolutionary tree construction

In this study, 68 completed genome maps of *Brucella melitensis* from the NCBI were used to construct an evolutionary tree based on the Genome Component Archive (GCA) registry number, and the genome assembly results were downloaded in fasta format, named with the GCA number. Single nucleotide polymorphism (SNP) calling and evolutionary tree construction were performed using kSNP (Version 4.1) (15) and *BM* bv.1 str. 16M was designated as the reference genome. The rest of the genomes were compared with this genome to analyze the whole genome-level SNPs. A total of 8,132 core SNPs were obtained from the 68 genomes used in the analysis. The kSNP nested algorithms were used to construct an evolution tree based on the core SNPs using the maximum likelihood method, and the evolution tree was visualized and displayed on the iTOL online platform (https://itol.embl.de/) (16).

### 2.9 Statistical analyses

All the data, including mice body weight, spleen weight, splenomegaly index, and the spleen bacterial load (CFU log10), were expressed as $\overset{\bar{\;}}{x}$ ± SEM (mean values ± standard errors of the mean) and analyzed by GraphPad Prism software (ver8.0, San Diego, CA). The unpaired t-test was used for comparison between the two groups. Repeated measures analysis of variance was employed, followed by the Tukey *post-hoc* analysis. *p* < 0.05 was used to determine statistical significance.

## 3 Results

### 3.1 The biological characteristics of *BM* CMCC55210a

**Figure 1:**
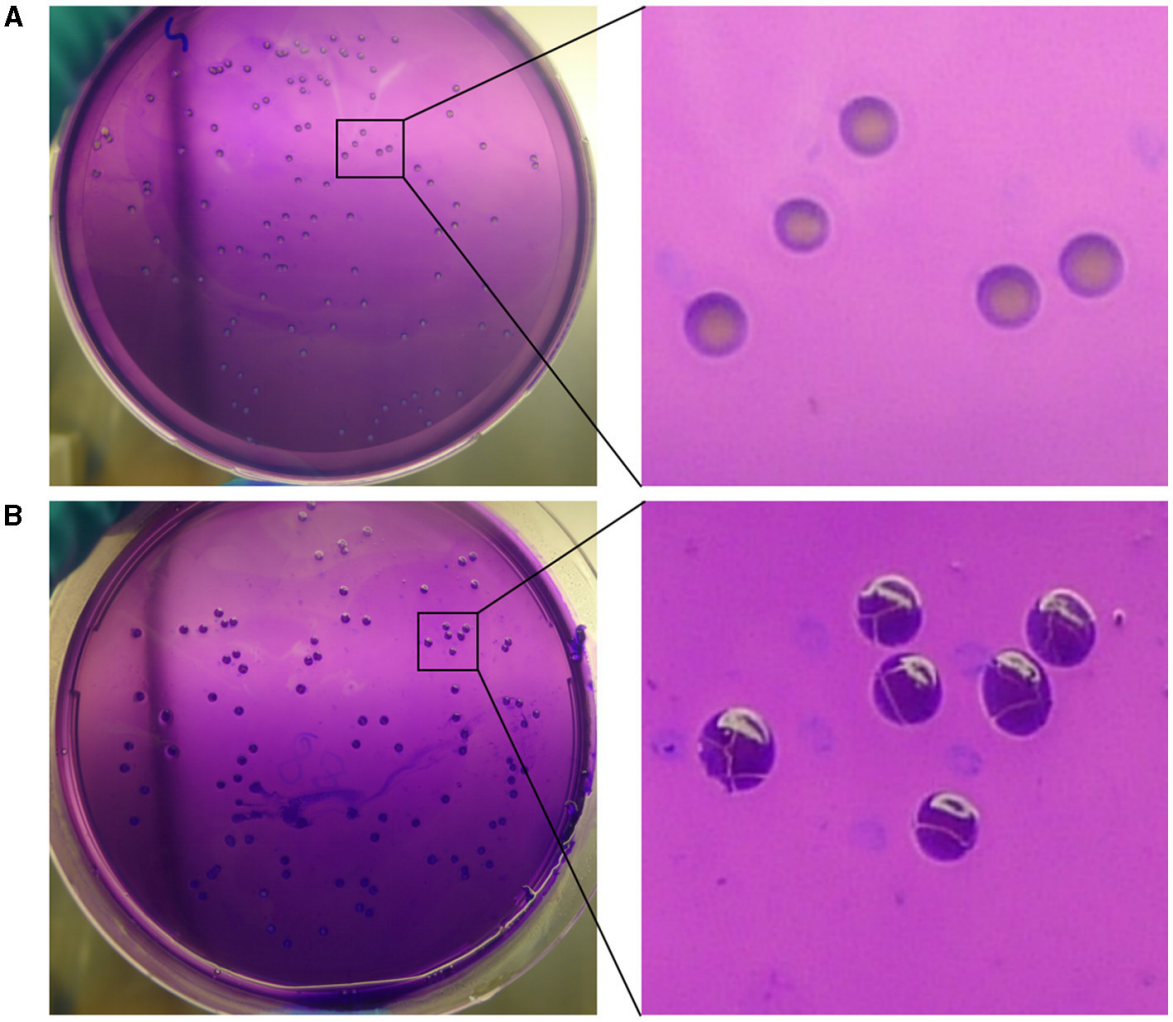
Crystal violet staining of *BM* CMCC55210 and CMCC55210a. **(A)** *BM* CMCC55210, **(B)** *BM* CMCC55210a. Colonies on TSA plates were examined by the naked eye. *BM* CMCC55210 settlements appeared light purple contrasted with the light violet background; CMCC55210a colonies seemed to be opaque deep purple under the light violet background.

In the mutation test assay for *BM* CMCC55210, we screened out one mutant colony from ~960 colonies tested, named CMCC55210a. To confirm whether the mutant strain originated from the virulent strain CMCC55210, we performed different biochemical reactions on CMCC55210a, including the dye-growth inhibition assay, the hydrogen sulfide-generating reaction assay, and the *Brucella* phage lysis assay. CMCC55210 and CMCC55210a grow well on the TSA plates with basic fuchsin and thionin added as typical TSA plates, and the lead acetate test papers stayed the same, indicating no hydrogen sulfide was produced during the cultivations of CMCC55210 and CMCC55210a. The *Brucella* bacteriophage BK2 lysed the strains of CMCC55210 and CMCC55210a, and the *Brucella* bacteriophages Tb, Wb, and Fi did not successfully lyse both strains. The above results showed that the biochemical characteristics of CMCC55210a were consistent with strain CMCC55210 (Table 1). Furthermore, the crystal violet staining assay showed that CMCC55210a colonies appeared to have a rough phenotype distinguished from the smooth CMCC55210 colonies (Figure 1). All the above results showed that the phenotypic variant strain CMCC55210a, derived from the parent strain CMCC55210, was a rough stable mutant strain.

**Table 1:** The biological characteristics of *BM* strains CMCC55210a and CMCC55210.

**Biological reactions**		***Brucella melitensis*** **strains**	
		**CMCC55210**	**CMCC55210a**
Growth on TSA containing (1:50,000 w/v)	Fuchsin	**+**	**+**
	Thionin	**+**	**+**
H_2_S production		–	**–**
Acriflavine agglutination		–	**+**
Crystal violet staining		–	**+**
Lysis by phage at RTD	Tb	–	–
	Wb	–	–
	Fi	–	–
	BK2	+	+
Phenotype		Smooth	rough

RTD, Routine Test Dilution; Tb, Tbilisi; Wb, Weybridge; Bk2, Berkeley; Fi, Firenze; **+**, positive; –, negative.

### 3.2 The *BM* CMCC55210a strain was attenuated

**Figure 2:**
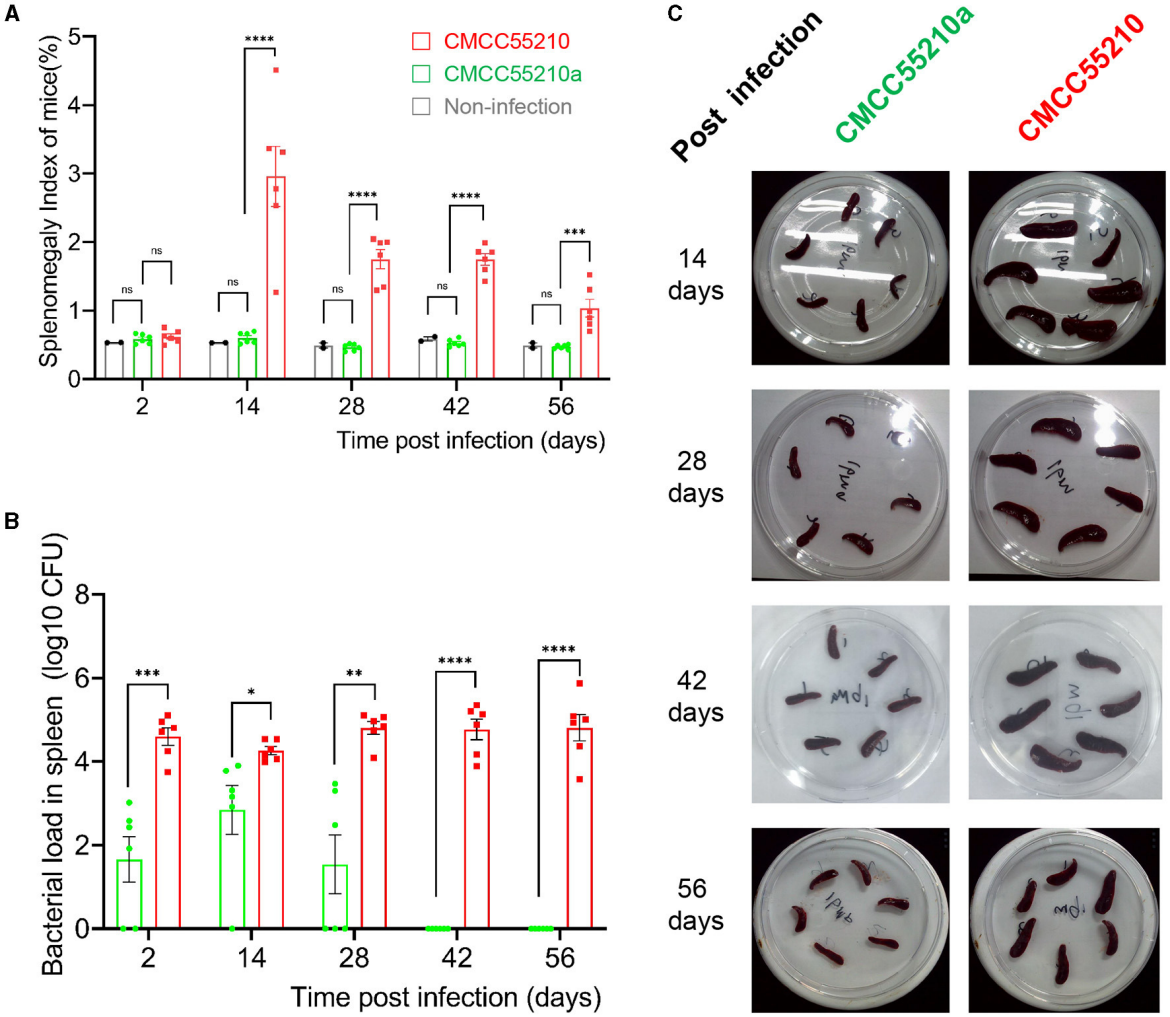
Virulence test of *BM* CMCC55210 and CMCC55210a by BALB/c mice infection. **(A)** Splenomegaly index of infected mice; **(B)** Log_10_ (CFU) of recovered from the spleen of infected mice; **(C)**. The gross spleen of mice infected by CMCC55210 and CMCC55210a. - ns, no significance; *p < 0.05; **p < 0.01; ***p < 0.001; ****p < 0.0001. Splenomegaly indexes and spleen gross morphology exhibited the same trend as the spleen weight **(A, C)**. The spleen size was approximately 5.0–8.0 × 3.0 mm of the CMCC55210a group in contrast to 10–15.0 × 3.0–5.0 mm of the *BM* CMCC55210 group **(C)**. The *Brucella* colonies recovered from the spleen (bacterial load) differed vastly. Approximately 104–106 bacteria were successfully cultured from the group of CMCC55210 over the 56 days. In comparison, 102–103 bacteria were detected from day 2 to 28 post-infection, and no bacteria were cultured from day 42 post-infection **(B)**.

To assess the virulence and the safety of the mutant strain CMCC55210a, a mice-infection model with intraperitoneal injection was used. First, BALB/c mice were challenged with an equal dose of 1.0 × 10^4^ CFU and 1.0 × 10^5^ CFU per mouse of the CMCC55210 and CMCC55210a strains in previous studies (17). At 14 and 28 days after the challenge, we sacrificed mice and determined spleen bacterial loads. The group of CMCC55210 infected mice had severely enlarged spleens with bacterial loads at 10^3^-10^5^ CFU in the spleen. In contrast, the group of CMCC55210a infected mice was mostly consistent with those of non-infected in the spleen size, and there was only one spleen with a challenging dose of 1.0 × 10^5^ CFU detected bacteria (Supplementary material). The results indicated that the challenging dose below 1.0 × 10^5^ CFU per mouse of CMCC5520a hardly caused spleen colonization in BALB/c mice. Furthermore, we used a high infection dose of CMCC55210 with an actual challenge dose of 1.50 × 10^5^ CFU per mouse (18) and CMCC55210a with an actual challenge dose of 5.60 × 10^7^ CFU per mouse (19, 20) to investigate the infection progression. Mice were sacrificed at 2, 14, 28, 42, and 56 days post-infection, and body weight, spleen weight, and spleen bacterial loads were determined. The mice's initial body weight was between 19 and 21 g and slowly gained to 23–24 g during the 56 days post-infection. There was no statistical significance among groups of non-infection, *BM* CMCC55210a and CMCC55210. The mice's spleen weight stayed the same between the group of non-infection and *BM* CMCC55210a, ~0.11–0.13 g over the 56 days, but the group of *BM* CMCC55210 mice's spleen weight was quite variable; the mean values were from 0.13 to 0.62, 0.41, 0.42, 0.25 at day 2, 14, 28, 42, and 56 post-infection. Correspondingly, splenomegaly indexes and spleen gross morphology exhibited the same trend as the spleen weight (Figures 2A, C). The spleen size was ~5.0–8.0 × 3.0 (mm) of the CMCC55210a group compared to 10.0–15.0 × 3.0–5.0 (mm) of the CMCC55210 group (Figure 2C). The spleen bacterial loads were vastly different. Approximately 10^4^-10^6^ *Brucella* bacteria were successfully cultured from the spleens of CMCC55210 infected mice group over 56 days, 10^2^-10^3^ bacteria after 2, 14, and 28 days, and no bacteria were detected after 42 days post-infection from the spleens of CMCC55210a infected mice (Figure 2B). The results of mice-infected experiments indicated that the virulence of the CMCC55210a strain attenuated in contrast to CMCC55210.

### 3.3 Evolutionary tree construction

The evolutionary tree showed that the strains of *BM* CMCC55210 and *BM* CMCC55210a in this study are the closest to the *BM* 16M strain in terms of evolutionary distance and co-clustered with the Rev.1 (passage 101) strain isolated from small ruminants in the United States in 1970 to form a separate branch (Figure 4). In contrast, strains NIPH_Bru1, NIPH_Bru4, NIPH_Bru9, NIPH_Bru11, NIPH_Bru24, and NIPH_Bru46, which were collected successively in Norway between 2003 and 2016, clustered into a separate dendrite, although the strains CMCC55210, CMCC55210a, 16M, and Rev.1 (passage 101) exhibit their independent evolution. *BM* BwIM_ITA_45 and *BM* BwIM_ITA_55, isolated successively in Italy in 2015 and 2016, are closely related to strain 2008724259, isolated in the United States in 2007, and to the ether (unknown source of isolation, place of isolation, and time of isolation) strain clustered into a separate branch. The other strains, in general, can be divided into two large clades, but the affinities are closer between strains within the clades. Different strains from the same geographical isolation area tend to be more similar. At the same time, there is no specific pattern, suggesting that the strains may not be significant in variation over different time spans.

### 3.4 Genome sequencing and comparative analysis of CMCC55210a and CMCC55210

#### 3.4.1 Genomic sequence characteristics

**Figure 3:**
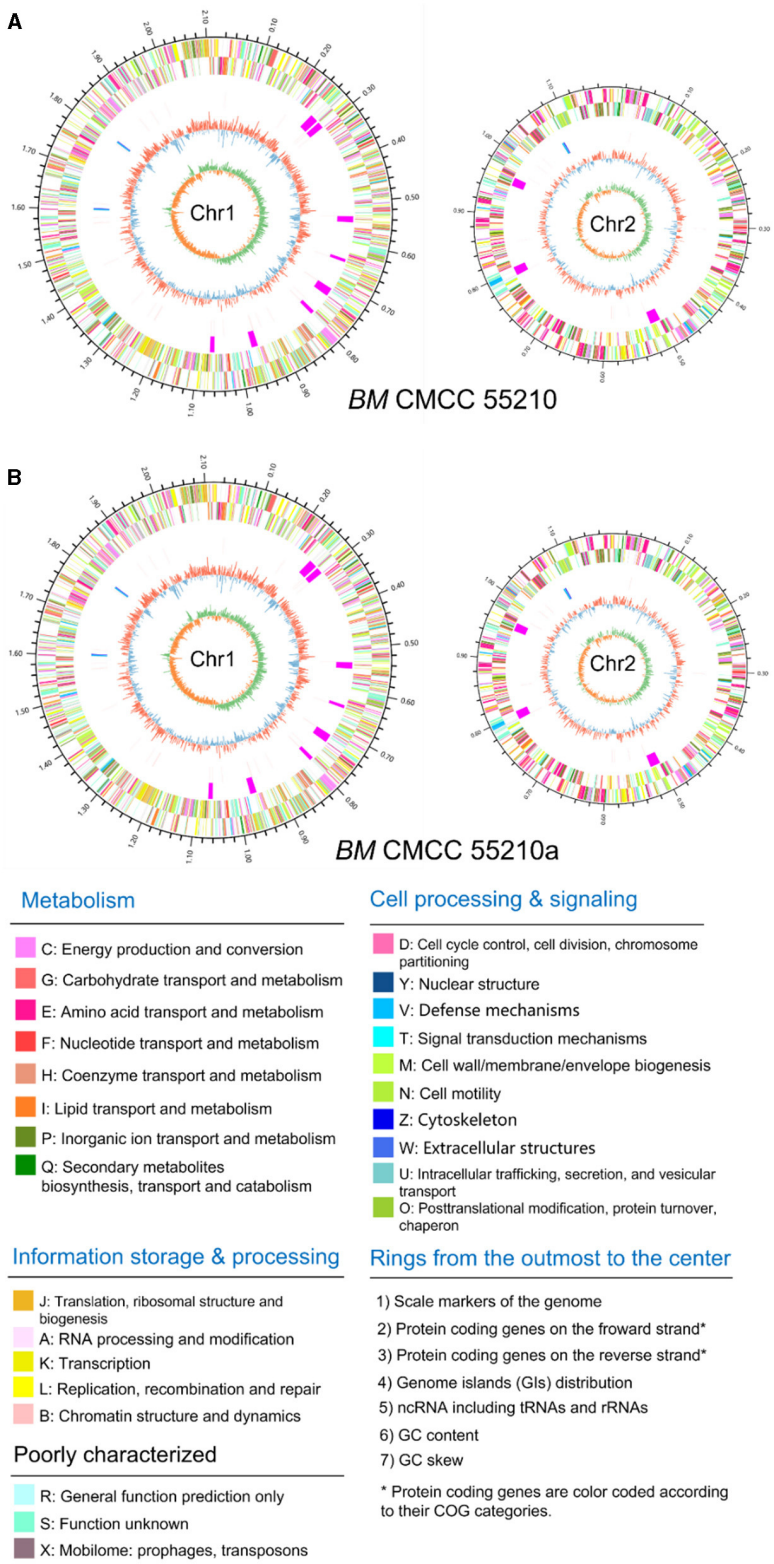
Circular genome maps of *BM* CMCC55210 chromosomes **(A)** and CMCC55210a chromosomes **(B)**. From the outer to the inner circle: (1) scale marks of genome; (2) assigned COG classes of protein-coding genes (CDSs) on the forward strand as indicated by relevant colors; (3) reverse strand CDSs; (4) genomic islands (GIs, fuchsia); (5) ncRNA including tRNA(red) and rRNA (16S, blue; 23S, celeste; 5S, green); (6) GC content (swell outward/inward indicates higher/lower G + C compared with the average G+C content); (7) GC skew (green/orange indicate positive/negative values).

Genomic details of the strain *BM* CMCC55210 were unclear, and the genome information of reference strain *BM* 16M (21) was generally used for analysis. Thus, we performed genome sequencing of both strains through Illumina and Pacbio platforms. The genomes of CMCC55210 and CMCC55210a were composed of two chromosomes; the lengths of Chr1 and Chr2 were 2.12 and 1.18 M, and the GC contents were 57.16 and 57.34%, respectively (Table 2). The average nucleotide identity between the two strains was 99.99%. Then, we performed COG function and genomic islands analysis within the genomes of CMCC55210 and CMCC55210a, and the results showed that there were no significant differences at the genomic level (Figure 3).

**Table 2:** Basic genome features of *BM* CMCC55210 and CMCC55210a.

**Features**	***BM*** **CMCC55210a**		***BM*** **CMCC55210**	
	**chr1**	**chr2**	**chr1**	**chr2**
Accession number	CP082107	CP082108	CP082109	CP082110
Length (bp)	2,117,101	1,177,797	2,117,097	1,177,809
G + C content	57.16%	57.34%	57.16%	57.34%
Predicted genes	2,173	1,176	2,173	1,175
Genes/genome	85.45%	87.88%	85.48%	87.88%
Average length (bp)	833	881	833	881
rRNAs	6	3	6	3
tRNAs	40	14	40	14
Prophage	4	0	4	0
Genome Islands	8	3	8	3

**Figure 4:**
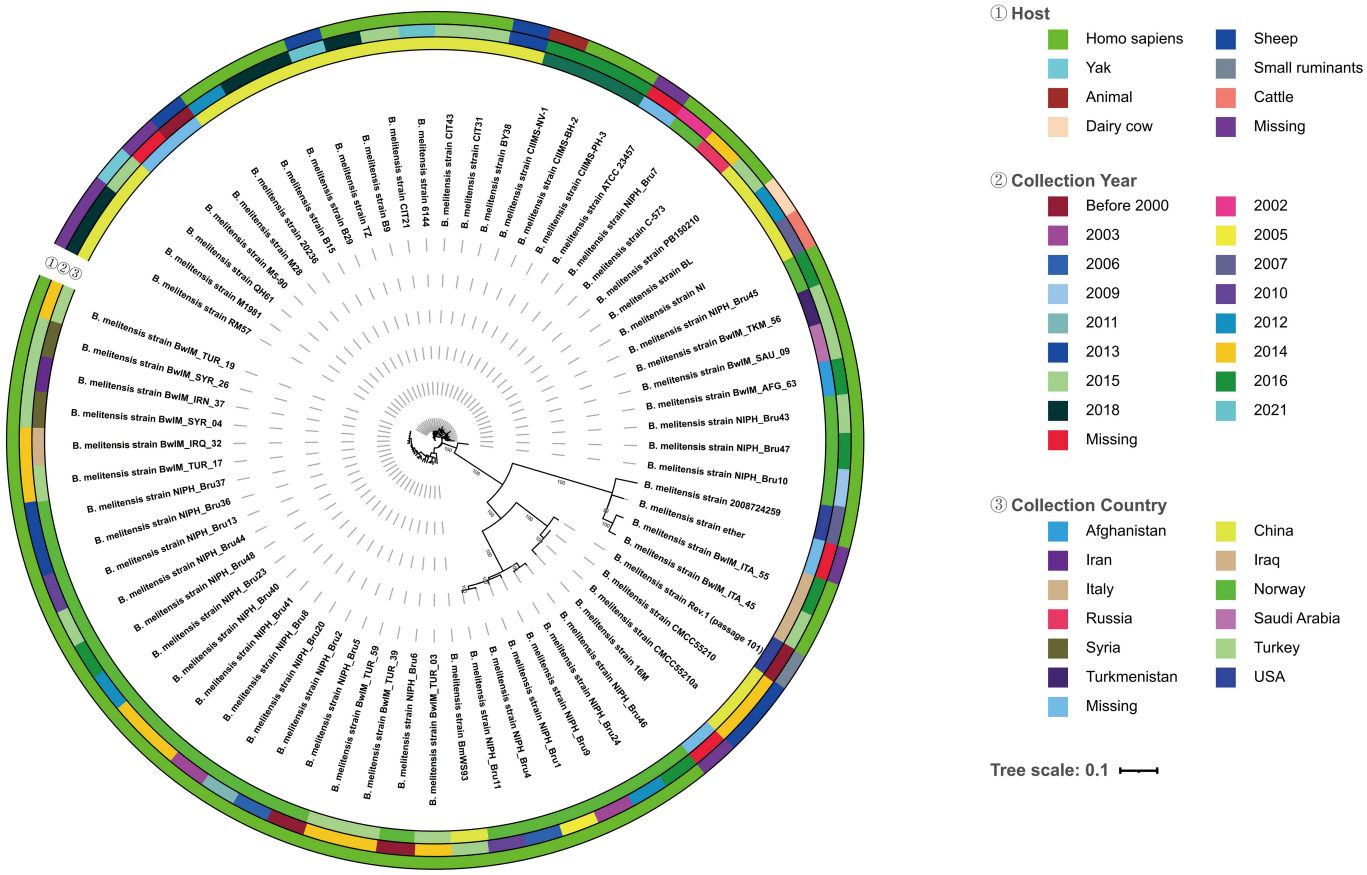
Evolutionary tree of 68 *Brucella melitensis* strains. From the outside to the inside of the tree, the first circle is the host information when the strain was isolated, and different colors represent different hosts; the second circle is the year when the strain was collected and isolated, and different colors represent different years; the third circle is the country where the original sample of the strain belongs to, and different colors represent different countries; the fourth circle is the strain information; the inner circle is the topology of the evolution tree; the inner circle is the topology of the evolution tree.

#### 3.4.2 Genome comparative analysis

To define genomic variations in mutant strain CMCC55210a, we performed comparative genomics between CMCC55210 and CMCC55210. The genome of CMCC55210 was used as analysis control, and 19 insertions (INs), 11 deletions (DELs), and 3 single nucleotide mutations occurred in the strain CMCC55210a. Considering the genome variations occurring in the intergenic region did not affect protein functions; therefore, we focused on mutations occurring in the protein-coding regions, including 4 INs, 8 DELs, and 2 single nucleotide mutations involving seven genes, namely *trmFO, trmD, xerC, pntA, hsdM, livM*, and *manB* (Table 3). From the gene length statistics, three genes, including the *xerC* (BRUCa_1896), *trmD* (BRUCa_1892), and *hsdM* (BRUCa_2973) of CMCC55210a strain, became shorter, whereas the *pntA* (BRUCa_3109) and *livM* (BRUCa_2789) were lengthened. The *manB* (BRUCa_2508) were the same length in all three samples, with only localized base and amino acid differences.

**Table 3:** Comparison and functional analysis of genes related to inner variations of *Brucella melitensis* CMCC55210a.

**Gene ID (NCBI) locus tag**	**Location on chromosome**	**Gene name**	**Gene variation among CMCC55210a, CMCC55210, and 16M from nucleotide level**				**Protein name CDS**	**Protein variations of CMCC55210**	**Biological function**	**References**
			**Nucleotide variation**	**55210a**	**55210**	**16M** **(****21****)**				
29593915 BME_RS05375	*Chr1*	*trmFO*	• 149C_del_ • 493G_in_ • 665G_in_	• *trmFO* • 1,401 bp	• *trmFO* • 861 bp	• *trmFO* • 1,401 bp	• TrmFO • 467 aa • WP002967605.1	TrmFO of 55210a is consistent with 16M	Folate/FAD-dependent tRNA (m(5)U54) methyltransferase, U54 modified to 5-methyluridine and forms a reverse Hoogsteen base pair with A58 that stabilizes the L-shaped tRNA structure	(22–24)
1629593725 BME_RS00705	*Chr1*	*trmD*	• 497, 498 • CA_del_	• *trmD1* • 1-519 • *trmD2* • 499–735	• *trmD* • 735 bp	• *trmD* • 735 bp	• TrmD • 245 aa • WP002964982.1	• Frameshift • TrmD1 1–173 aa, TrmD2 167–245 aa • TrmD of 55210a • terminates at 173 aa and an altered peptide sequence from position 166	• TrmD catalyzes the N(1)-methylguanosine (m(1)G) modification at p37 in tRNAs with the (36)GG(37) • The m(1)G37-modified tRNA functions properly to prevent +1 frameshift errors on the ribosome	(25–28)
29594142 BME_RS00695	*Chr1*	*xerC*	• 160C_del_ • G256C • (A → P) • 328C_in_	• *xerC1* • 1–183 • *xerC2* • 241-948	• *xerC* • 948 bp	• *xerC* • 948 bp	• XerC • 316 aa • WP004684445.1	• Frameshift • XerC1 1–61 aa • XerC2 81–316 aa • split into two incomplete ORFs	• The XerCD site-specific recombination is involved in the stable inheritance of circular replicons • xerC also contributes to biofilm-associated infections and acute bacteremia due to agr-independent and -dependent pathways in MRSA	(29–34)
29595944 *BME_RS11770*	*Chr2*	*pntA*	• 1125G_del_	• *pntA* • 1,239 bp	• *pntA* • 1,218 bp	• *pntA* • 1,218 bp	• PntA • 406 aa • WP004682363.1	Frameshift, change in its amino acid sequence from position 376 and extension of the peptide chain	PntA is the Re/Si-specific NAD(P)(+) transhydrogenase subunit alpha, PntAB functions to balance the NADH: NADPH equilibrium specifically in the direction of NADPH	(34, 35)
29595445 BME_RS12390	*Chr2*	*hsdM*	• 716A_del_ • 788A_del_ • 1072C_del_	• *hsdM1* • 1–747 • *hsdM2* • 818-1093 • *hsdM3* • 1087–1527	• *hsdM* • 1,527 bp	• *hsdM* • 1,527 bp	• HsdM • 509 aa • WP002965802.1	• Frameshift, truncated and broken HsdM • HsdM1 1–249 aa, • HsdM2 273–345 aa, • HsdM3 363–509 aa	Class I SAM-dependent DNA methyltransferase, type I restriction endonucleases methylate host DNA with hsdS. related with virulence and induce drug resistance on *Mycobacterium tuberculosis*	(36–42)
79382350 *BME_RS18385*	*Chr2*	*livM*	• 789G_in_	• *livM* • 1,002 bp	• *livM* • 849 bp	• *livM* • 450 bp	• LivM • 150 aa • WP011005660.1	Frameshift, extension of the peptide chain	Branched chain amino acid, phenylalanine ABC transporter membrane subunit, permease	(43, 44)
29595165 *BME_RS14595*	*Chr2*	*manB*	• 1067T → C (L356P)	• *manB* • 1,434 bp	• *manB* • 1,434 bp	• *manB* • 1,434 bp	• ManB • 478 aa • WP002966241.1	Point mutation, mutation of leucine to proline at position 356	Phosphomannomutase, involved in o-ps biosynthesis of LPS	(45–47)

#### 3.4.3 *xerC*

**Figure 5:**
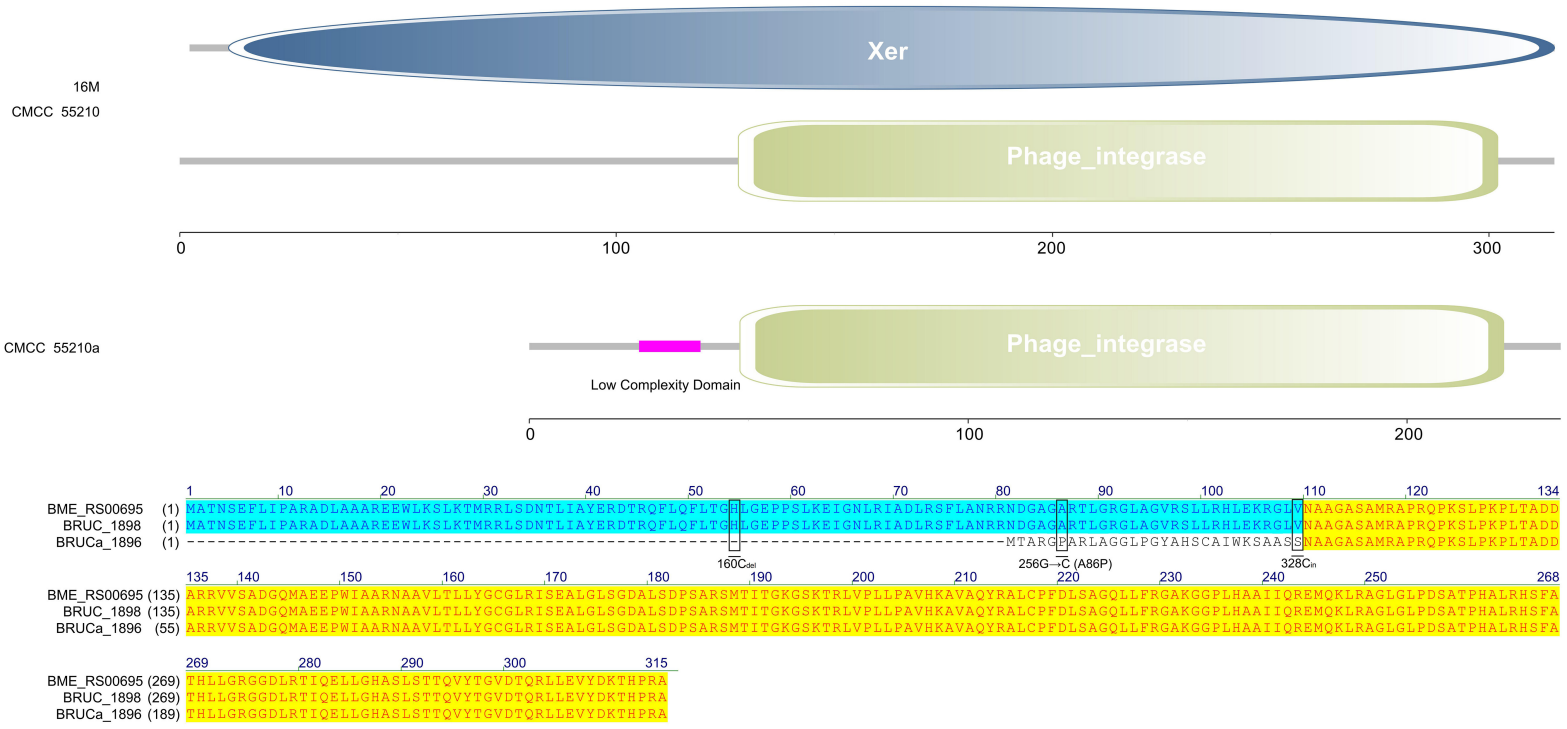
Functional domains predicted of XerC and protein sequence alignment. The XerC sequences of strains 16M (BME_RS00695) and CMCC55210 (BRUC_1898) were identical. They contained two large functional domains, Xer and Phage integrase. The *xerC* (BRUCa_1896) in strain CMCC55210a had a deletion of cytosine **(C)** at site 160, resulting in a shortening of the 5′ end of the gene. Other variations occurred in xerC of CMCC55210a, including guanine (G) to C at site 256, resulting in the change of amino acid from Alanine (A) to Proline (P) and the insertion of C at site 328.

The protein XerC sequences of strains 16M (BME_RS00695) and CMCC55210 (BRUC_1898) were identical. They contained two large functional domains, Xer and Phage integrase, whereas the corresponding gene, BRUCa_1896, in strain CMCC55210a, had a deletion of cytosine (C) at site 160, resulting in a shortening of the 5′ end of the gene. Other variations occurred in *xerC* of CMCC55210a, including guanine (G) to C at site 256, resulting in the change of amino acid from alanine (A) to proline (P) and the insertion of C at site 328 (Figure 5). The most critical variant may be the incomplete Xer functional domain affecting the gene's normal function. The XerCD site-specific recombination system is important for the stable inheritance of circular replicons, such as plasmids and bacterial chromosomes (29). Similar to other bacteria, *Brucella* species harbor circular chromosomes. The formation of chromosome dimers is caused by odd numbers of crossovers during homologous recombination (30). Translocase FtsK activates XerCD recombination (31, 32) at a specific site, *dif*, so that recombination occurs at the right time and place. Recent studies revealed that XerD of *Staphylococcus aureus* and *Bacillus subtilis* unloads structural maintenance of chromosome (SMC) complexes through the binding of XerD to additional chromosomal loci, not *dif*, a different action that does not depend on XerC (33). We hypothesize that the redundancy mechanism of XerC-XerD also exists in *Brucella* to ensure bacterial chromosome stabilization.

#### 3.4.4 *trmFO*

**Figure 6:**
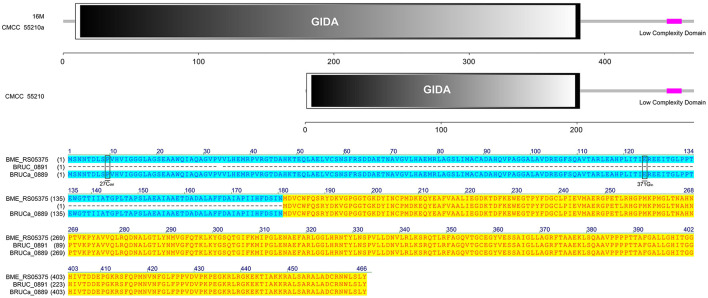
Functional domains predicted of TrmFO and protein sequence alignment. The TrmFO sequences were identical in strains 16M and CMCC55210a, and both contained complete GIDA functional domains. There was a cytosine deletion at 27 and a cytosine insertion at 371 in gene *trmFO* (BRUC_0891) of the CMCC55210 strain, which resulted in an incomplete GIDA functional domain.

The protein TrmFO sequences of strains 16M and CMCC55210a were identical, and both contained complete GIDA functional domains. In contrast, the corresponding gene BRUC_0891 in CMCC55210 strain was unable to form a normal functional protein due to a code-shift mutation at the 5′ end of the gene because of a deletion of a base C at the 27th base and an insertion of a base C at the 371st base, which resulted in the formation of normal functional proteins, and a 180 amino acid deletion at the 5′ end of the gene relative to the proteins in the other two samples, which resulted in an incomplete GIDA functional domain. It thus affected the regular expression and functional roles of the protein (Figure 6).

There is a 5-methyluridine at position 54 (T54) in the T-loop of bacteria tRNAs, and T54 methylation can stabilize the tRNA structure. The methyl group is transferred through two pathways: (1) from S-adenosylmethionine by TrmA methyltransferase (22) and (2) from 5,10-methylenetetrahydrofolate by TrmFO, a folate/FAD-dependent methyltransferase (23). In most Gram-positive and several Gram-negative bacteria, TrmFO orthologs have been identified through sequence analyses (24). TrmA orthologs, a class I SAM-dependent RNA methyltransferase, were found in *Brucella* species. In our study, gene *trmFO* of *BM* CMCC55210a was consistent with *BM* 16M. The mutations that occurred in *trmFO* may not be associated with the attenuation mechanisms.

#### 3.4.5 *trmD*

**Figure 7:**
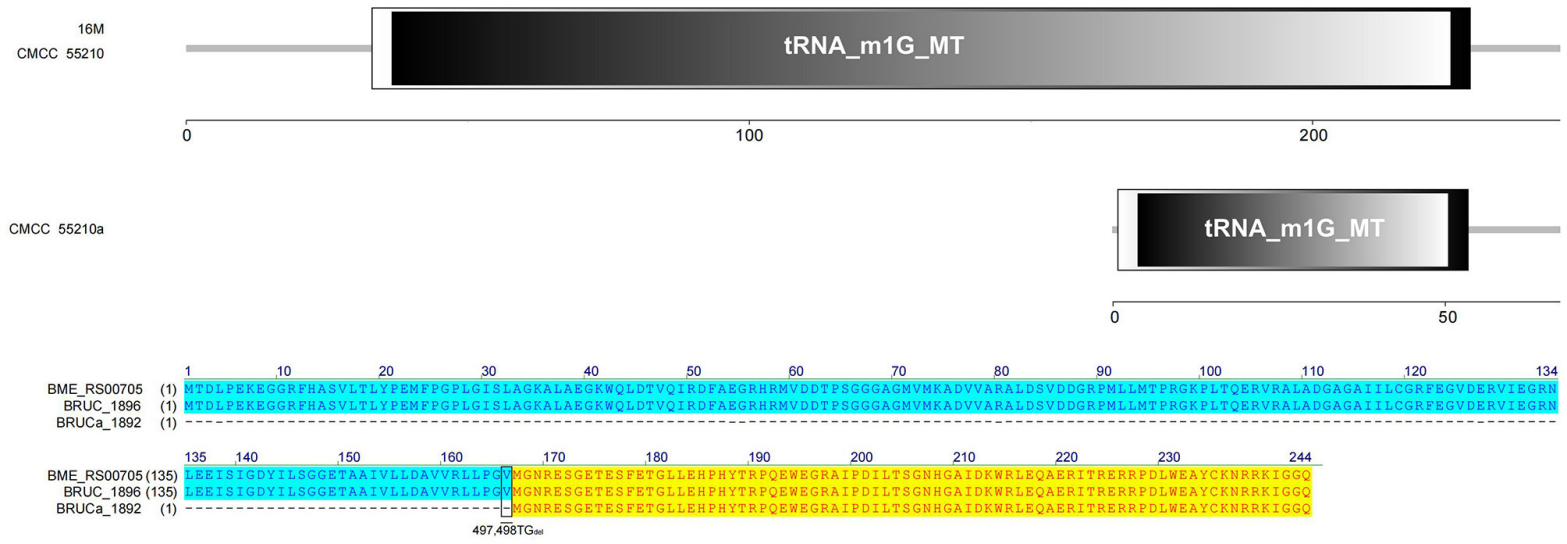
Functional domains predicted of TrmD and protein sequence alignment. The TrmD sequences of *BM* 16M and CMCC55210 were identical with the complete tRNA_m1G_MT functional domain. The *trmD* (BRUCa_1892) of CMCC55210a had a shifted mutation due to the deletion of two bases, T and G, at positions 497 and 498, which resulted in an incomplete tRNA_m1G_MT functional domain.

The TrmD protein, encoded by the gene *trmD*, is a tRNA methyltransferase that suppresses translational frameshift errors at proline codons through methylation modification of tRNA m1G37, which is essential for bacterial growth (25, 26). The process of methylation modification exerted in *Haemophilus influenzae* by TrmD requires stabilization of S-adenosyl-L-methionine (AdoMet) at position 171 by phenylalanine (Phe) and capture of the tRNA 38-position phosphate group by glycine (Gly) at position 59 (27). Protein alignment analysis suggested that the TrmD corresponding active site of *BM* CMCC55210 was consistent with *Haemophilus influenzae*. The protein TrmD sequences of *BM* 16M and CMCC55210 were identical, and both contained the complete tRNA_m1G_MT functional domain. In contrast, the corresponding gene BRUCa_1892 in *BM* CMCC55210a had a shifted mutation due to the deletion of two bases, T and G, at positions 497 and 498, which resulted in a shifted mutation of the gene and a shortening of the 5′ end, leading to an incomplete tRNA_m1G_MT functional domain, which in turn affected the regular expression and functional role of the protein (Figure 7). This mutation is considered lethal to bacteria, but our results showed that the growth of *Brucella melitensis* CMCC55210a was not affected.

Another evolutionary study found that cumulative mutations were detected in the gene encoding proline-tRNA ligase (proline-tRNA ligase, *proS*) of restored growth of *Escherichia coli trmD* mutant strains (28). ProS amplifies m1G37 by distinguishing between m1G37 modified and unmodified tRNA^Pro^ to safeguard the translational accuracy, thereby strengthening the effects. The aminoacylation efficiency of m1G37-unmodified tRNA^Pro^ was lower than that of m1G37-modified tRNA^Pro^, and the restored growth was detected in *trmD* mutant strains by the aminoacylation of m1G37-unmodified tRNA^Pro^. Furthermore, sequence alignments revealed that the *proS* nucleotide sequence of *BM* CMCC55210a was unchanged, so unknown potential regulatory mechanisms that helped CMCC55210a adapt to the effects of gene *trmD* mutation need to be investigated.

#### 3.4.6 *hsdM*

**Figure 8:**
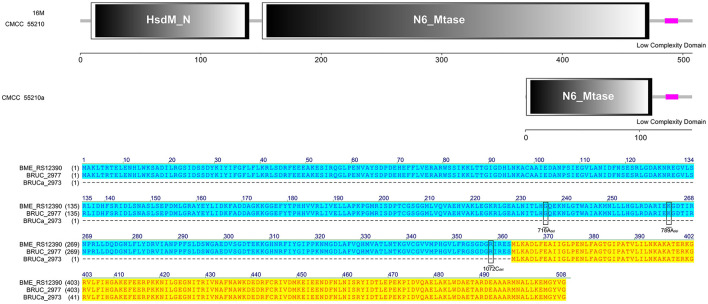
Functional domains predicted of HsdM and protein sequence alignment. The HsdM sequences of strain 16M and CMCC55210 were identical, containing complete functional domains of HsdM_N and N6_Mtase. The hsdM (BRUCa_2973) in CMCC55210a with three deletions of 716A_del_, 788A_del_, and 1072C_del_, which resulted in HsdM_N domain lost and truncated N6_Mtase domain.

The protein HsdM sequences of strain 16M and CMCC55210 samples were identical, containing complete functional domains of HsdM_N and N6_Mtase. In contrast, there are three deletions of 716A_del_, 788A_del_, and 1072C_del_ in the corresponding gene BRUCa_2973 of *BM CMCC55210a* (Figure 8). The deletion of the bases led to the coding gene shifting. The 5′ end is shortened, the HsdM_N functional domain is lost, and the N6_Mtase functional domain is incomplete, thus affecting the regular expression and functional role of the protein. HsdM, a methyltransferase (MTase) encoded by gene *hsdM*, is a subunit of type I restriction–modification (R-M) systems that provides a major defense against phages and invasive DNA in bacteria (36, 37). Typical R-M systems are composed of three separate subunits of DNA-specificity (HsdS), DNA-modification (HsdM), and DNA restriction (HsdR) (38, 39). Another new type I R-M system can utilize two copies of HsdM and one HsdS to form the M2-S MTase complex, one of which has NPPF in the highly conserved catalytic motif IV and modifies adenine to m6A, and one having an NPPY catalytic motif IV and modifying cytosine to m4C (40). Mutations in gene *hsdM* were detected in *Streptococcus suis* serotype 2 attenuated strains, but it was unclear whether HsdM mutations were associated with reduced resistance to phagocytosis (40). So, the relationship between *hsdM* and the attenuation of *BM* CMCC55210a in this study also needs to be further verified.

#### 3.4.7 *livM*

**Figure 9:**
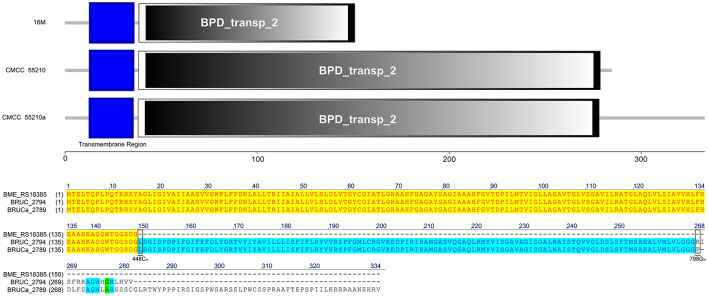
Functional domains predicted of LivM and protein sequence alignment. The LivM protein sequences of strains 16M, CMCC55210, and CMCC55210a are not identical. The BPD_transp_2 domain was incomplete because of the *livM* (BME_RS18385) of 16M with 448C_del_. *BM* CMCC55210a had an extended sequence due to 798G_in_. The BPD_transp_2 structural domains of the strain CMCC55210 and CMCC55210a (amino acid from 38 to 278) are different at the end of the 3′ end, but both are structurally complete.

The LivM protein sequences of strains 16M, CMCC55210, and CMCC55210a are not identical. At site 448, that is, before the last triplet codon of the gene of the 16M sample, a single C is inserted, which results in a shifted mutation, the disappearance of the original termination codon, and a lengthening of the gene sequence. At site 798, *BM* CMCC55210a inserts a single G relative to CMCC55210, thus allowing the gene sequence to continue to be extended and the total protein sequence is the longest of the three. In terms of structural domains, the transmembrane region structural domains at the 5′ end were all complete. The sequences were 100% identical, while the BPD_transp_2 structural domain, strain 16M gene, was incomplete, with a portion of the 3′ end missing. The BPD_transp_2 structural domains of the strain CMCC55210 and CMCC55210a (position from amino acid 38–278) are different at the 3′ end, but both are structurally complete (Figure 9). The mutation of the CMCC55210a sample gene at the 3′ end may affect the protein's tertiary structure or active ability, which in turn affects the protein's function. Leucine-isoleucine-valine (LIV) is the major transport system in bacteria for the branched-chain amino acids. The *livM*-encoded protein localized to the inner membrane of the bacteria cell is one of the membrane components LivHMGF, that is required for high-affinity leucine transport (43, 44). At least six copies of *LivHMGF* operons were detected in the chromosomes of *BM* CMCC55210a and CMCC55210. We supposed the mutation that occurred in *livM* of CMCC55210a had no relationship with the rough phenotype.

#### 3.4.8 *pntA*

**Figure 10:**
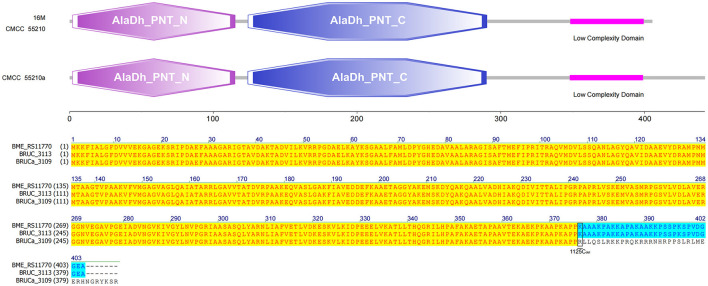
Functional domains predicted of PntA and protein sequence alignment. The PntA sequences of 16M and CMCC55210 were identical. The *pntA* (BRUCa_3109) of CMCC55210a with 1125C_del_ resulted in a shifted mutation that affects the low complexity domain differently.

The gene *pntA* of all three strains contains three functional domains. The protein sequences of strains 16M and CMCC55210 were identical. The gene BRUCa_3109 corresponding to strain CMCC55210a is missing a single C at site 1125, resulting in a shifted mutation that affects the third functional domain differently. The third functional domain, low complexity, is located from amino acid 348 to amino acid 399 in the protein sequences corresponding to all three samples (Figure 10). The gene *pntA* encodes PntA, which is the α subunit of the pyridine nucleotide transhydrogenase (PntAB). As a catalyzer of energy-coupled electron transfers between NAD(H) and NADP(H), PntAB regulates electron carrier redox balance (35). Previous studies have demonstrated that pntAB adaptive truncation mutation to decrease PntAB-catalyzed NADPH biosynthesis occurred in *E. coli* K-12 MG1655 phosphoglucose isomerase-deficient strain with enhancing environmental adaptability through adaptive evolution (34).

#### 3.4.9 *manB*

**Figure 11:**
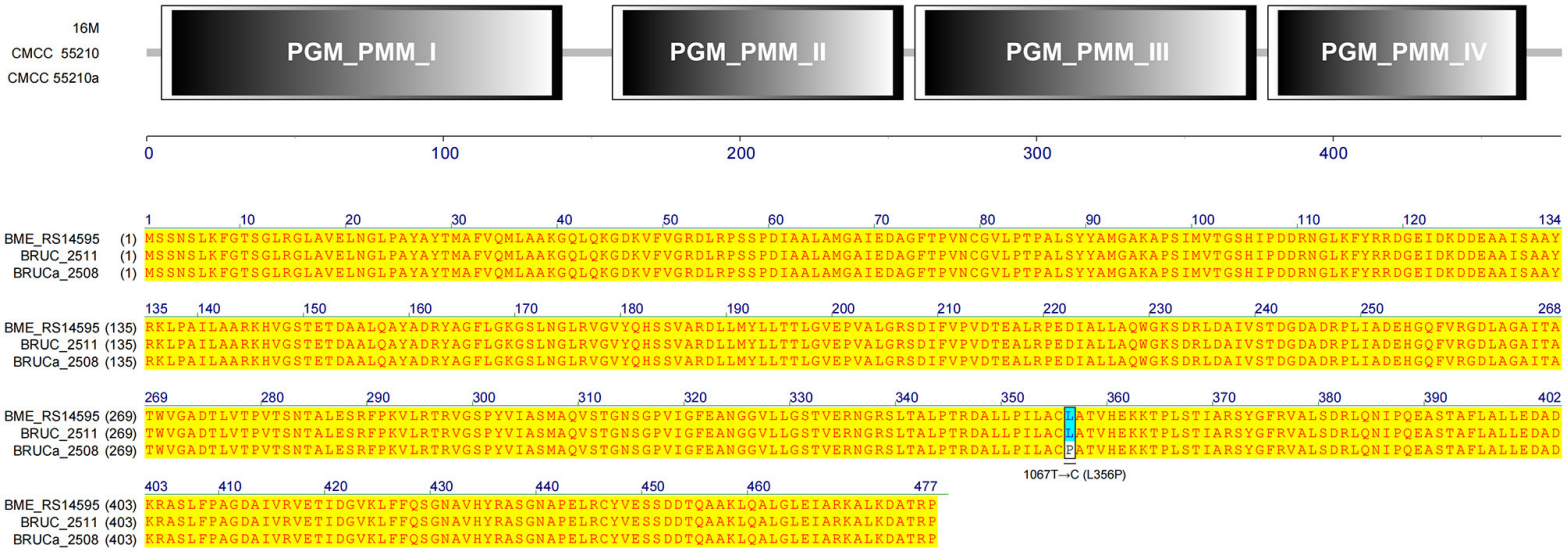
Functional domains predicted of ManB and protein sequence alignment. The gene *manB* was identical in sequence length in all three strains. The corresponding gene BRUCa_2508 in strain CMCC55210a has an SNP mutation at 1067 from thymine (T) to guanine (C), which results in L356P in the functional domain PGM_PMM_III.

The gene *manB* was identical in sequence length in all three strains. The corresponding gene BRUCa_2508 in strain CMCC55210a has an SNP mutation at 1067 from thymine (T) to guanine (C), which results in a change in the coding amino acid from leucine (L) to proline (P) and the functional domain corresponding to this mutation position is PGM_PMM_III (Figure 11). Phosphomannomutase (ManB), encoded by *manB*, converts mannose-6-phosphate to mannose-1phosphate, which is a key metabolic factor during the synthesis of lipopolysaccharide (LPS) (45). LPS is composed of three domains: lipid A, core oligosaccharide, and O-antigen (46). The lack of the O-antigen or core oligosaccharide contributes to the rough LPS phenotype of *Brucella melitensis*. It has been found that *manB*, interrupted by the transposon mutant strain of *Brucella melitensis* H38, confronted attenuation and lack of the core oligosaccharide and the O-antigen (47). Proline is the most common first residue of α-helix and the marginal chain of β-sheet, a disruptor in the secondary structure of the protein owing to its unique ring-loading structure of the side chain that maintains exceptional conformational rigidity (48). We hypothesized that the L356P mutation may affect the structure of α-helices 12, thereby affecting the spatial conformation of the glycan-binding region of domain PGM_PMM_III. The crystal violet staining experiment showed that CMCC55210a was a rough strain, and its rough change might be caused by the truncated LPS structure.

#### 3.4.10 Diagram of key genomic differences and their mechanistic implications for bacteria–host interactions

**Figure 12:**
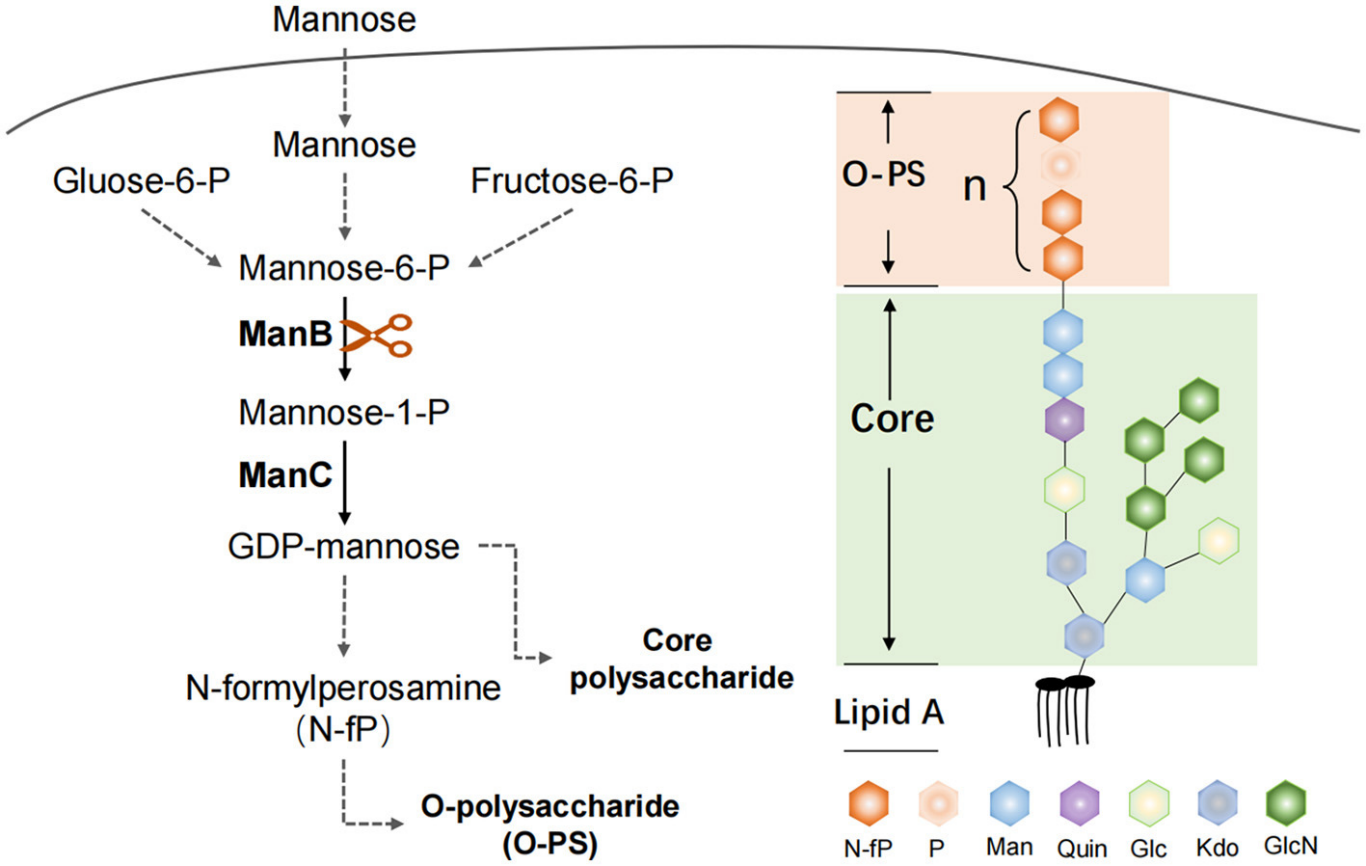
ManB function in the *Brucella* LPS synthesis. The protein ManB as phosphomannomutase converts mannose-6-phosphate to mannose-1phosphate, which is a key metabolic factor during the synthesis of lipopolysaccharide (LPS). LPS consists of three parts: Lipid A, core polysaccharide, and O-antigen polysaccharide (O-PS). Crippled LPS exhibits a rough phenotype.

We summarize a brief map of key genomic differences and their mechanistic implications for bacteria–host interactions in Figure 12.

## 4 Discussion

*Brucella* spp. are facultative intracellular pathogens that have the ability to survive and multiply in professional and non-professional phagocytes and cause abortion in domestic animals and undulant fever in humans. Several species are recognized within the genus *Brucella*, and this classification is mainly based on the difference in pathogenicity and host preference. *Brucella* strains may occur as either smooth or rough, expressing smooth LPS (S-LPS) or rough LPS (R-LPS) as major surface antigens. This bacterium possesses an unconventional non-endotoxic lipopolysaccharide that confers resistance to antimicrobial attacks and modulates the host immune response. The genome sequences of the *B. melitensis, B. suis*, and *B. abortus* became available. The genomes of *B. suis, B. melitensis*, and *B. abortus* are very similar in sequence, organization, and structure. Few fragments are unique among the genomes. Although many aspects of its biology remain to be understood, the sequencing and annotation of its genome paved the way for a highly comprehensive and rapid analysis of its proteome.

Vaccination and intensive diagnostics are the primary approaches for brucellosis control worldwide (49, 50). The lack of human vaccines and the problems with veterinary vaccines, such as abortion in pregnant animals, have made the development of ideal vaccines that are highly immunogenic, safe, and provide long-lasting protection a hot topic (51). New vaccines with higher safety, such as carrier, subunit, peptide, DNA, and nanoparticle, are constantly being developed and have shown good protective ability in mouse or guinea pig infection experiments. However, they also have problems, so large-scale vaccination is still dominated by live attenuated vaccines in practical applications. Among the various vaccine development strategies, live attenuated vaccines with some virulence genes inactivated seem to be more effective, such as M5-90Δ26 for sheep and A19-ΔVirB12 for cattle, which are recommended in the Chinese Brucellosis Control Guidelines, and at the same time provide help in distinguishing between natural infection and immune-protection by serologic examination.

Several studies on virulence factors were directed at the main components of the outer membrane. The outer membrane contains lipopolysacharides (LPS), which is the *Brucella* major virulence factor. The LPS was identified as a major virulence determinant of *Brucella* and recognized for its role in virulence when naturally occurring isolates lacking LPS showed reduced survival. In this study, we hypothesized that two mutations occurred in attenuated strain CMCC55210a associated with its virulence changes. One is the point mutation in the manB gene, which is related to LPS synthesis in the previous studies. The manB mutant strains result in a large part of LPS deletion and present the rough phenotype, such as the M5-90ΔmanB strain, which shows high protection by mice infection experiment (52). The LPS was identified as a major virulence determinant of *Brucella* and recognized for its role in virulence when naturally occurring isolates lacking LPS showed reduced survival. The hydrophobic lipid A region constitutes mostly the outer coating of the outer membrane and is responsible for many of the endotoxic properties attributed to LPS. Thermotropic phase behavior and immunochemical analysis of *B. abortus* and *B. melitensis* lipid A suggest a disaccharide backbone molecule linked in a β1–6 configuration. Nevertheless, our study only demonstrated reduced virulence and easy clearance *in vivo* with mice infection experiments. We did not examine the LPS structure of the strain CMCC55210a in depth compared with CMCC55210 and did not determine the protective ability of the strain CMCC55210a as a vaccine strain. Our results show that attenuated strain CMCC55210a has potential as a vaccine strain.

The type I restriction–modification (RM) system contains three host specificity determinant (hsd) genes coding a modification subunit (M), which protects the host DNA through DNA methylation by methyltransferase activity; a restriction subunit (R), which digests the foreign DNA by restriction endonuclease activity; and a specificity subunit (S), which determines the recognition sequence of both restriction and modification activities by the central repeat region and two target recognition domains (TRDs). Type I restriction endonucleases can influence the composition and dynamics of the gut microbiota by providing certain bacterial strains with a competitive advantage. Bacteria equipped with specific R–M systems can protect themselves against invading foreign DNA, potentially altering the balance of microbial species within the gut environment. Another mutation in gene hsdM might affect the function of the R–M system and the methylation from the whole bacteria level. Studies showed that hsdS contributed to bacterial anti-phagocytosis and survival in adverse host environments by positively impacting the transcription of two peptidoglycan-binding protein genes, enhancing resistance to reactive oxygen species, and reducing the secretion of TNF-α and nitric oxide by phagocytes. Different strains with broken R-M systems showed different virulence changes (36, 53–55). We hypothesize that the mutation occurring in subunit HsdM of strain CMCC55210 assists virulence reduction.

Current vaccines such as S19 and RB51 for cattle are live attenuated strains, which have proven effective but have limitations, including residual virulence in humans and interference with diagnostic tests. Future strategies may involve the development of safer, genetically modified live attenuated vaccines that maintain immunogenicity but have reduced virulence and are distinguishable from wild-type strains in diagnostic tests. This opens up a new possible strategy for screening ideal vaccines by disrupting the function of the bacterial R-M system in conjunction with targeted LPS synthesis-related gene mutations.

## 5 Conclusion

We obtained a spontaneously rough strain named CMCC55210a, which evolved from the smooth strain *BM* CMCC55210. Biochemical identification tests and *in vivo* experiments in mice verified the strain to be stable and attenuated. Through comparative genomic analysis, a possible mechanism of virulence reduction from single nucleotide mutation in phosphomannomutase encoding gene manB was shown at the gene level. Contributing to understanding the complex relationship between pathogens, the gut microbiome, and the immune system, it also opens avenues for innovative therapeutic interventions in inflammatory diseases driven by microbial and immune dysregulation.

## Data availability statement

The original contributions presented in the study are included in the article/Supplementary material, further inquiries can be directed to the corresponding author.

## Ethics statement

The animal study was reviewed and approved by Animal Welfare and Ethics Committee of the School of Basic Medical Science of Fudan University (Approval No. 20201105-001).

## Author contributions

WH: Conceptualization, Methodology, Writing—original draft. DW: Conceptualization, Formal analysis, Methodology, Writing—original draft. ZS: Data curation, Software, Writing—original draft. DQ: Project administration, Supervision, Writing—review & editing.
